# Evaluation of retinal and choroidal microvascular changes in patients who received hydroxychloroquine by optical coherence tomography angiography

**DOI:** 10.5935/0004-2749.20210001

**Published:** 2025-02-02

**Authors:** Esat Cinar, Berna Yuce, Fatih Aslan

**Affiliations:** 1 Ekol Eye Hospital, Izmir, Turkey; 2 Ophthalmology Clinic, İzmir Tepecik Training and Research Hospital, University of Health Sciences, Izmir, Turkey; 3 Department of Ophthalmology, Alaaddin Keykubat University, Alanya, Turkey

**Keywords:** Retina/drug effects, Choroid/drug effects, Optical coherence tomography, Hydroxychloroquine, Fluorescein angiography/methods, Retina/efeitos dos fármacos, Coroide/efeitos de fármacos, Tomografia de coerência óptica, Hidroxicloroquina, Angiofluoresceinografia/métodos

## Abstract

**Purpose:**

The aim of the study is to evaluate the retinal and choroidal microvascular changes via optical coherence tomography angiography in patients who received hydroxy chloroquine.

**Methods:**

In total, 28 eyes of 28 patients (24 females, and 4 males) receiving treatment with hydroxychloroquine were assessed in this cross-sectional cohort study (hydroxychloroquine group). The high-and low-risk groups consisted of patients receiving hydroxychloroquine for ≥5 years (14 eyes of 28 patients) and <5 years (14 eyes of 28 patients), respectively. A total of 28 ageand gender-matched volunteers were enrolled as the control group. The macular flow area (superficial, deep, and choriocapillaris), superficial and deep vessel density, foveal avascular zone area, central foveal thickness, and subfoveal choroidal thickness parameters were measured by optical coherence tomography angiography.

**Results:**

The mean age of the 28 patients who received hydroxychloroquine and the 28 age-matched controls was 45.5 ± 11.1 years (range: 29-70 years) and 44.5 ± 13.9 years (range: 28-70 years), respectively. In patients who received hydroxychloroquine, the values for the superficial, deep, and choriocapillaris macular flow areas were 13.578 ± 0.30, 13.196 ± 0.31, and 17.617 ± 0.42, respectively. In controls, these values were 16.407 ± 0.95, 13.857 ± 0.31, and 18.975 ± 0.76, respectively (p<0.05 for all). The superficial, deep, and chor iocapillaris flow areas were significantly smaller in patients who received hydroxychloroquine than those in controls (p<0.05 for all). Superficial and deep vessel densities were significantly reduced in patients who received hydroxychloroquine in all regions (i.e., foveal, parafoveal, temporal, superior, nasal, and inferior) (p<0.05 for all). Moreover, significant difference was observed between the groups in the foveal avascular zone area (superficial and deep), central foveal thickness, and subfoveal choroidal thickness (p<0.05 for all).

**Conclusions:**

Retinochoroidal microvascular flow and vessel density of the macular area were significantly decreased in patients who received hydroxychloroquine. Hyd roxychloroquine may damage the retinochoroidal micro vascular architecture. Optical coherence tomography angiography may contribute to the early detection of hyd roxychloroquine-induced retinal toxicity.

## Abstract (pt)

**Objetivo:**

O objetivo do estudo foi de avaliar as alterações microvasculares da retina e da coroide em pacientes sob hidroxicloroquina, através da angiografia por tomografia de coerência óptica.

**Métodos:**

Este é um estudo transversal de coorte que avaliou um total de 28 olhos de 28 pacientes (24 mulheres e 4 homens) submetidos a tratamento com hidroxicloroquina (grupo da hidroxicloroquina). Catorze olhos de 28 pacientes em uso de hidroxicloroquina por mais de 5 anos foram definidos como sendo o grupo de alto risco, ao passo que o grupo de baixo risco consistiu em 14 olhos de 28 pacientes em uso de hidroxicloroquina por menos de 5 anos. Foram ainda incluídos 28 voluntários como grupo de controle, pareados por idade e sexo. Através de angiografia por tomografia de coerência óptica, foram medidos os seguintes parâmetros: área do fluxo macular (superficial, profundo e coriocapilar), densid ade vascular superficial e profunda, área da zona avascular foveal e espessura da coroide subfoveal.

**Resultados:**

Foram recrutados para o estudo um total de 28 pacientes sob tratamento com hidroxicloroquina, com idade média de 45,5 ± 11,1 (29-70) anos, e 28 membros do grupo de controle, pareados por idade e sexo, com idade média de 44,5 ± 13,9 (28-70) anos. As áreas superficial, profunda e coriocapilar do fluxo macular foram respectivamente de 13,578 ± 0,30, 13,196 ± 0,31 e 17,617 ± 0,42 nos pacientes em tratamento com hidroxicloroquina e, respectivamente de 16,407 ± 0,95, 13,857 ± 0,31 e 18,975 ± 0,76 no grupo de controle (p<0,05 para todos os valores). As três medições de área do fluxo macular foram significativamente menores nos pacientes em uso de hidroxicloroquina em comparação com os indivíduos do grupo de controle (p<0,05 para todos os valores). As densidades vasculares superficial e profunda mostraram-se significativamente reduzidas em todas as regiões (foveal, parafoveal, temporal, superior, nasal e inferior) nos pacientes em uso de hidroxicloroquina (p<0,05 para todos os valores). Finalmente, também foi observada uma diferença significativa entre os grupos em relação à área da zona avascular foveal (superficial e profunda), à espessura foveal central e à espessura da coroide subfoveal (p<0,05 para todos os valores).

**Conclusão:**

O fluxo microvascular retinocoroidal e a densidade vascular da área macular mostraram-se significativamente diminuídos nos pacientes sob hidroxicloroquina. Este fármaco pode danificar a arquitetura microvascular retinocoroidal e a angiografia por tomografia de coerência óptica pode contribuir para a detecção precoce da toxicidade retiniana induzida pela hidroxicloroquina.

## INTRODUCTION

Hydroxychloroquine (HCQ) is an antimalarial drug, which is widely used in rheumatology and dermatology clinics for the treatment of numerous autoimmune diseases^(1)^. However, there is hesitation among clinicians regarding its use due to its irreversible retinal toxicity^(2)^. Although the mechanism involved in this process remains unclear, its destructive effect on the retinal pigment epithelium (RPE), photoreceptors, and retinal ganglion cell-inner plexiform layer complex due to its affinity to melanin pigment has been demonstrated^(3-6)^. The effect of HCQ on the vascular structure and its role in retinal toxicity are unclear. Analysis of the superficial and deep retinal vascular layers became possible only recently, and the data concerning this topic are newly presented.

Avoidance of the irreversible visual loss related to HCQ-induced retinal toxicity is crucial for the detection of retinal toxicity prior to the onset of RPE damage^(7)^. In their revised protocol in 2016, the American Academy of Ophthalmology stated that the visual field test, optical coherence tomography (OCT), multifocal electroretinogram (ERG), microperimetry, and fundus autofluorescence may be used as needed to screen the retinal toxicity in patients who receive HCQ^(5)^.

OCT angiography (OCTA) is a new, non-invasive method that allows the evaluation of the superficial and deep flow and vessel density of the macula^(8-10)^.

The aim of this study is to compare the retinal superficial capillary plexus (SCP), deep capillary plexus (DCP), choroidal thickness, and foveal avascular zone (FAZ) area between patients who received HCQ and healthy subjects by OCTA imaging.

## METHODS

### Participants

Twenty-eight eyes from 28 patients (24 females and 4 males) who received HCQ and 28 sexand age-matched controls were enrolled in the study. The study protocol was approved by Alanya Alaaddin Keykubat University School of Medicine Clinical Researches Ethics Committee (N^o^ 2-17/2019). The research adhered to the tenets of the Declaration of Helsinki, and detailed written informed consent was provided by all individuals prior to their participation in the study.

### Study design

This was a cross-sectional cohort study. Patients who had a history of continuous treatment with HCQ for ≥12 months and ongoing treatment with HCQ (200 mg/day) were included in our study group. There was no restriction applied for the maximum duration of treatment with HCQ, and all participants who fulfilled the minimum criteria were included. One eye (randomly selected) of each patient was analyzed in both the study and control groups.

The high- and low-risk groups consisted of patients receiving HCQ for ≥5 years (14 eyes of 28 patients) and <5 years (14 eyes of 28 patients), respectively. A total of 28 age-and sex-matched volunteers, selected from patients who presented to the ophthalmology outpatient clinic for routine ophthalmologic examination, were enrolled as the control group.

Exclusion criteria for all participants were as follows: nystagmus; corneal opacity; cataract; glaucoma; congenital or acquired retinal disorders, including any vascular disease; or a history of ocular trauma or surgery. Individuals with any systemic disease (except rheumatoid arthritis, Sjögren’s syndrome, connective tissue disease, and systemic lupus erythematosus), including diabetes mellitus, arterial hypertension, anemia, renal disease, and cardiovascular disease, were excluded. In addition, participants who had a history of any chronic drug use, including analgesics, antihistamines, vasodilators, decongestants, anticoagulants, oral contraceptives, and sildenafil, were excluded.

### Examination

Age, systolic blood pressure, and diastolic blood pressure were recorded. A comprehensive ophthalmic examination included the following: best-corrected visual acuity assessment using the Snellen chart; slit-lamp anterior segment examination; axial length measurement by the IOLMaster device (ver. 3.02; Carl Zeiss, Meditec, Jena, Germany); intraocular pressure measurements by Goldmann applanation tonometry; dilated fundus examination with a 90-D lens, central 10° visual field test using Octopus 900 (Interzeag AG, Schlieren-Zurich, Switzerland); and OCTA measurement (RT Vue XR100-2; Optovue Inc., Fremont, CA, USA). The retinochoroidal structure in all individuals was evaluated using OCTA. All OCTA scans were performed in the morning (between 10:00 a.m. and 12:00 p.m.) to avoid diurnal fluctuations.

### OCTA measurements

Optovue Angio-Vue system technology (Software Version 2015.1.1.98; Optovue Inc.) allows for quantitative analysis. The inner and outer boundaries for SCP were assumed to be 3 µm below the internal limiting membrane and 16 µm below the inner plexiform layer, respectively. The inner and outer boundaries were 16 and 70 µm below the inner plexiform layer for DCP, respectively. The outer retina was located 70 and 30 µm below the inner plexiform layer and the RPE, respectively^(11-13)^. The OCTA software automatically outputs the flow area value. The vessel density was separately calculated in five regions (i.e., fovea, temporal, superior, nasal, and inferior) based on the Early Treatment Diabetic Retinopathy Study contour. A 3 × 3 mm macular angiogram of the choriocapillaris (CC) layer was analyzed using the Optovue software with flow function to measure the CC flow area^(13)^. The flow area of CC was calculated automatically as vessel areas of CC divided by selected areas. FAZ and central foveal thickness are measured automatically using OCTA. Subfoveal choroidal thickness (SFCT) is defined as the distance between the hyper-reflective line corresponding to the base of the RPE and the hyper-reflective line corresponding to the chorio-scleral interface. It was measured thrice by two independent observers using manual calipers in the horizontal and vertical sections beneath the fovea. Average values were recorded and analyzed.

### Statistical analysis

One eye from each participant was randomly selected for analysis using the SPSS for Windows version 21.0 (IBM Corp., Armonk, NY, USA) software. This selection was based on the absence of a significant difference between the right and left eyes. The simple randomization technique of computer-generated random numbers was used to select the eyes. For each continuous variable, normality was determined using the Kolmogorov-Smirnov test, which showed a normal distribution for all parameters. The categorical variables were analyzed using the chi-squared test. OCTA measurements of the groups were compared using the independent t-test. Spearman correlation analysis was applied between macular perfusion parameter data and daily and cumulative doses in the HCQ group. Statistically significant differences are denoted by p-values <0.05.

## RESULTS

This cross-sectional study analyzed 28 eyes of 28 patients who received treatment with HCQ and 28 eyes of 28 age-and sex-matched controls. There were no significant differences observed between the study and control groups in terms of age, spherical equivalent, axial length, systolic blood pressure, diastolic blood pressure, visual acuity, and intraocular pressure parameters. Demographic data, clinical diagnosis, mean daily dose, cumulative drug dose, and mean duration of treatment are shown in table 1.

**Table 1:** The demographic and clinical characteristics of the HCQ and control groups

Characteristic	HCQ group (n=28)	Control group (n=28)	p
Age (mean, years)	45.5 ± 11.1	44.5 ± 13.9	0.935
SBP (mmHg)	113.75 ± 5.3	115.61 ± 5.1	0.901
DBP (mmHg)	77.1 ± 4.7	75.8 ± 3.1	0.788
IOP (mmHg)	14.2 ± 1.3	13.9 ± 1.2	0.915
SE (D)	0.232 ± 0.41	0.224 ± 0.43	0.762
AL (mm)	23.17 ± 0.68	23.19 ± 0.49	0.947
BCVA (Snellen)	0.621 ± 0.74	0.648 ± 0.68	0.503
Cummulative drug dose (g)	593.714 ± 450	-	-
Duration of drug use (months)	63 ± 11.2 (12-132)	-	-
Daily dose (mg/day)	292.857 ± 85 (200-400)	-	-
**Systemic diseases**			
Rheumatoid arthritis	5		
Sjögren’s syndrome	5		
Connective tissue disease	8		
Systemic lupus erythematosus	10		

Values are presented as the mean ± SD.

HCQ= hydroxychloroquine; SE= spherical equivalent; AL= axial length; SBP= systolic blood pressure; DBP= diastolic blood pressure; IOP= intraocular pressure; BCVA= best-corrected visual acuity; SD= standard deviation.

**Figure 1:**
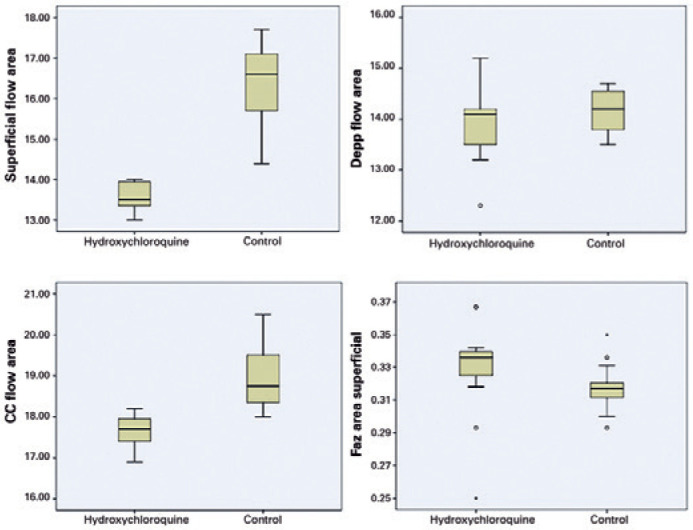
The boxplot analysis representing the macular flow area measurements (superficial, deep, and CC) and FAZ area for both groups.

The macular flow area, including the superficial retinal flow area, deep flow area, and CC, was significantly smaller in HCQ-treated patients than that in controls (p<0.05 for all) (Table 2). Moreover, the FAZ area was significantly enlarged in the HCQ group versus that in the control group (superficial: p=0.034 and deep: p=0.013) (Table 2). The boxplot analysis representing the macular flow area measurements (superficial, deep, and CC) and FAZ area for both groups is shown in figure 1.

**Table 2:** Macular perfusion and perimetric parameters of the HCQ and control groups

Parameter	HCQ group n=28	Control group n=28	P
Superficial retinal flow area (mm^2^)	13.578 ± 0.30	16.407 ± 0.95	0.001^*^
Deep retinal flow area (mm^2^)	13.196 ± 0.31	13.857 ± 0.31	0.012^*^
CC flow area (mm^2^)	17.617 ± 0.42	18.975 ± 0.76	0.002^*^
FAZ area (superficial, mm^2^)	0.331 ± 0.014	0.310 ± 0.018	0.034^*^
FAZ area (deep, mm^2^)	0.357 ± 0.010	0.309 ± 0.018	0.013^*^
Superficial vessel density (%)			
Fovea	34.053 ± 1.83	36.635 ± 1.22	0.013^*^
Parafoveal	53.520 ± 1.27	55.771 ± 1.28	0.011^*^
Temporal	54.135 ± 0.97	56.292 ± 1.21	0.014^*^
Superior	53.678 ± 1.36	56.760 ± 0.86	0.002^*^
Nasal	53.496 ± 0.61	56.492 ± 1.00	0.002^*^
Inferior	53.910 ± 0.94	56.428 ± 1.06	0.001^*^
Deep vessel density (%)			
Fovea	36.157 ± 0.71	37.978 ± 0.55	0.032^*^
Parafoveal	55.446 ± 1.01	57.285 ± 0.56	0.030^*^
Temporal	54.903 ± 1.66	56.685 ± 1.07	0.025^*^
Superior	54.956 ± 1.41	57.214 ± 0.58	0.016^*^
Nasal	52.635 ± 0.93	56.225 ± 0.93	0.023^*^
Inferior	52.867 ± 1.01	55.575 ± 1.08	0.015^*^
Central foveal thickness	236.783 ± 3.8	244.829 ± 4.2	0.044^*^
Subfoveal choroidal thickness	308.099 ± 9.2	322.082 ±11.47	0.041^*^
Perimetry			
Mean defect (dB)	2.84 ± 1.79	0.93 ± 0.37	0.027^*^
Standard loss variance (dB)	1.92 ± 0.85	0.62 ± 0.39	0.013^*^

Values are presented as the mean ± SD.

* = statistically significant.

HCQ= hydroxychloroquine; CC= choriocapillaris; FAZ= foveal avascular zone; SD= standard deviation.

CC= choriocapillaris; FAZ= foveal avascular zone.

Superficial and deep vessel densities were significantly reduced in HCQ-treated patients for all macular regions (i.e., foveal, parafoveal, temporal, superior, nasal, and inferior) (p<0.05 for all) (Table 2).

When compared with those in the healthy controls, central foveal thickness and SFCT in HCQ-treated patients were significantly decreased (p=0.044 and 0.041, respectively) (Table 2).

The perimetric data (10° visual field test) were evaluated using two parameters; both the mean defect (dB) and standard loss variance (dB) were significantly increased in the HCQ group (Table 2).

Spearman correlation analysis between macular perfusion parameters (superficial, deep, and CC flow area), FAZ area, perimetric data, and daily and cumulative doses in the HCQ group showed weak statistical significance. However, the duration of disease was not correlated with any of those parameters. Table 3 presents the results of Spearman correlation analysis.

**Table 3:** Correlation analysis with the disease duration, cumulative dose, and the duration of drug use

		Duration of disease				Cumulative dose			Duration of drug use		
		**p**	**r**		**p**		**r**	**p**		**r**	
Superficial retinal flow area (mm^2^)		0.695	-0.078		0.001^*^		-0.001	0.000^*^		-0.730	
Deep retinal flow area (mm^2^)		0.280	0.211		0.045^*^		-0.471	0.000^*^		-0.550	
CC flow area (mm^2^)		0.978	-0.001		0.032^*^		-0.521	0.022^*^		-0.121	
FAZ area (superficial, mm^2^)		0.067	0.351		0.046^*^		0.144	0.034^*^		0.185	
FAZ area (deep, mm^2^)		0.071	0.414		0.041^*^		0.213	0.021^*^		0.431	
	Superficial vessel density (%)										
	Fovea	0.951	0.012		0.023^*^		-0.230	0.041^*^		-0.160	
	Parafovea	0.978	0.005		0.034^*^		-0.187	0.040^*^		-0.123	
	Temporal	0.840	0.040		0.016^*^		-0.560	0.013^*^		-0.501	
	Superior	0.435	0.154		0.033^*^		-0.191	0.014^*^		-0.441	
	Nasal	0.381	-0.172		0.025^*^		-0.222	0.013^*^		-0.291	
	Inferior	0.335	-0.189		0.006^*^		-0.797	0.001^*^		-0.798	
Deep vessel density (%) Fovea		0.259	-0.221		0.029^*^		-0.203	0.020^*^		-0.299	
Parafovea		0.123	-0.298		0.036^*^		-0.178	0.010^*^		-0.513	
Temporal		0.512	-0.129		0.045^*^		-0.104	0.029^*^		-0.207	
Superior		0.885	-0.029		0.028^*^		-0.510	0.029^*^		-0.452	
Nasal		0.402	-0.165		0.028^*^		-0.156	0.037^*^		-0.246	
Inferior		0.795	-0.051		0.001^*^		-0.811	0.003^*^		-0.638	
Central foveal thickness		0.381		0.172	0.037^*^		-0.287	0.012^*^		-0.239	
Subfoveal choroidal thickness		0.196		0.174	0.043^*^		-0.285	0.036^*^		-0.301	
Perimetry Mean defect (dB)		0.004		0.508	0.001^*^		0.797	0.001^*^		0.852	
Standard loss variance (dB)		0.003		0.602	0.001^*^		0.678	0.001^*^		0.924	

P-values are presented with Spearman’s correlation coefficient tests.

* = statistically significant CC= choriocapillaris; FAZ= foveal avascular zone.

Patients were divided into two subgroups based on the duration of treatment with HCQ: high-risk group (duration of treatment ≥5 years) and low-risk group (duration of treatment <5 years). Comparison between the high- (14 patients) and low-risk (14 patients) groups revealed statistically significant differences in terms of superficial retinal flow area, deep retinal flow area, CC flow area, and FAZ area (p<0.05) (Table 4). Moreover, the measurement of superficial and deep vessel densities in the HCQ group revealed significant differences between the high-and low-risk groups in all macular regions (i.e., foveal, parafoveal, temporal, superior, nasal, and inferior) (p<0.05 for all) (Table 4).

**Table 4:** Macular perfusion and perimetric parameters of the highand low-risk groups

	High-risk group	Low-risk group	P
Superficial retinal flow area (mm^2^)	13.674 ± 0.33	14.221 ± 0.85	0.021^*^
Deep retinal flow area (mm^2^)	13.258 ± 0.37	14.114 ± 0.21	0.032^*^
CC flow area (mm^2^)	16.987 ± 0.44	17.955 ± 0.46	0.024^*^
FAZ area (superficial, mm^2^)	0.330 ± 0.018	0.311 ± 0.22	0.033^*^
FAZ area (deep, mm^2^)	0.328 ± 0.018	0.308 ± 0.011	0.025^*^
Superficial vessel density (%) Fovea	34.167 ± 1.63	35.835 ± 1.12	0.023^*^
Parafovea	53.540 ±1.17	54.111 ± 1.37	0.028^*^
Temporal	53.935 ± 0.97	55.311 ± 1.87	0.013^*^
Superior	53.278 ± 1.38	55.360 ± 0.45	0.013^**^
Nasal	53.444 ± 0.52	54.687 ± 1.52	0.028^*^
Inferior	53.463 ± 0.81	55.328 ± 1.24	0.010^*^
Deep vessel density (%) Fovea	36.211 ± 0.78	36.978 ± 0.55	0.042^*^
Parafovea	55.101 ± 1.21	56.962 ± 0.55	0.038^*^
Temporal	53.803 ± 1.45	55.289 ± 1.37	0.015^*^
Superior	54.384 ± 1.28	56.814 ± 0.84	0.025^*^
Nasal	52.666 ± 0.90	55.125 ± 0.87	0.022^*^
Inferior	52.966 ± 1.10	54.324 ± 1.33	0.027^*^
Central foveal thickness	223.241 ± 9.5	231.829 ± 4.2	0.033^*^
Subfoveal choroidal thickness	311.997 ± 9.8	319.821 ± 11.47	0.047^*^
Perimetry Mean defect (dB)	2.72 ± 1.81	2.21 ± 1.21	0.041^*^
Standard loss variance (dB)	1.65 ± 0.75	1.62 ± 0.39	0.845^*^

Values are presented as the mean ± SD.

* = statistically significant. CC= choriocapillaris; FAZ= foveal avascular zone.

The correlation between OCTA parameters and visual acuity was assessed in the highand low-risk groups. There was no correlation detected between the OCTA parameters and visual acuity in either group (p>0.005, for all).

Patients treated daily with ≥6.5 mg/kg HCQ (high-risk for retinopathy) and <6.5 mg/kg HCQ (low-risk for retinopathy) were compared. Significant differences were observed in all locations (foveal, parafoveal, temporal, superior, nasal, and inferior) both in the SCP and DCP layers of the macula between the highand low-risk groups. Furthermore, visual field parameters were worse in the high-risk group than those in the low-risk group, and the difference was statistically significant (details shown in table 5).

**Table 5:** Macular perfusion and perimetric parameters of analysis with lower doses versus higher doses in patients treated with HCQ

	Lower dose (<6.5 mg/kg daily) n=16	Higher dose (>6.5 mg/kg daily) n = 12	P
Superficial retinal flow area (mm^2^)	15.333 ± 0.97	13.189 ± 0.48	0.001^*^
Deep retinal flow area (mm^2^)	13.423 ± 0.44	13.001 ± 0.59	0.043^*^
CC flow area (mm^2^)	18.211 ± 0.70	17.859 ± 0.84	0.032^*^
FAZ area (superficial, mm^2^)	0.304 ± 0.018	0.300 ± 0.010	0.039^*^
FAZ area (deep, mm^2^)	0.348 ± 0.017	0.365 ± 0.02	0.043^*^
Superficial vessel density (%)			
Fovea	36.031 ± 0.92	35.214 ± 0.98	0.044^*^
Parafoveal	54.456 ±1.17	53.274 ±1.15	0.023^*^
Temporal	55.951 ± 1.34	55.001 ± 1.27	0.034^*^
Superior	55.276 ± 0.99	54.102 ± 1.19	0.042^*^
Nasal	55.853 ± 1.24	54.267 ± 1.40	0.021^*^
Inferior	55.3789 ± 1.12	55.007 ± 1.07	0.045^*^
Deep vessel density (%)			
Fovea	36.249 ± 0.59	35.308 ± 0.96	0.026^*^
Parafoveal	56.173 ± 0.65	55.179 ± 0.93	0.020^*^
Temporal	55.127 ± 1.84	54.178 ± 1.37	0.018^*^
Superior	56.851 ± 0.79	55.164 ± 0.63	0.039^*^
Nasal	55.441 ± 1.10	54.227 ± 1.07	0.037^*^
Inferior	54.012 ± 1.22	53.278 ± 1.32	0.024^*^
Central foveal thickness	240.257 ± 4.9	238.521 ± 5.12	0.054
Subfoveal choroidal thickness	318.002 ±11.88	311.542 ± 12.21	0.030^*^
Perimetry			
Mean defect (dB)	0.88 ± 0.33	0.90 ± 0.22	0.043^*^
Standard loss variance (dB)	0.51 ± 0.44	0.54 ± 0.17	0.026^*^

Values are presented as the mean ± SD.

* = statistically significant

HCQ= hydroxychloroquine; CC= choriocapillaris; FAZ= foveal avascular zone; SD= standard deviation.

In addition, we compared data from the low-risk group and healthy control group. The low-risk group exhibited significantly lower superficial and deep vessel densities compared with the healthy controls (details are shown in table 6). Parameters for both eyes of HCQ-treated patients are presented in table 7.

**Table 6:** Macular perfusion and perimetric parameters of the low-risk and healthy control groups

	Low-risk group n=14	Control group n=28	P
Superficial retinal flow area (mm^2^)	14.221 ± 0.85	16.407 ± 0.95	0.013^*^
Deep retinal flow area (mm^2^)	14.114 ± 0.21	13.857 ± 0.31	0.509
CC flow area (mm^2^)	17.955 ± 0.46	18.975 ± 0.76	0.044^*^
FAZ area (superficial, mm^2^)	0.311 ± 0.22	0.310 ± 0.018	0.665
FAZ area (deep, mm^2^)	0.308 ± 0.011	0.309 ± 0.018	0.711
Superficial vessel density (%)			
Fovea	35.835 ± 1.12	36.635 ± 1.22	0.043^*^
Parafovea	54.111 ± 1.37	55.771 ± 1.28	0.041 ^*^
Temporal	55.311 ± 1.87	56.292 ± 1.21	0.041 ^*^
Superior	55.360 ± 0.45	56.760 ± 0.86	0.022^*^
Nasal	54.687 ± 1.52	56.492 ± 1.00	0.023^*^
Inferior	55.328 ± 1.24	56.428 ± 1.06	0.028^*^
Deep vessel density (%)			
Fovea	36.978 ± 0.55	37.978 ± 0.55	0.046^*^
Parafovea	56.962 ± 0.55	57.285 ± 0.56	0.103
Temporal	55.289 ± 1.37	56.685 ± 1.07	0.024^*^
Superior	56.814 ± 0.84	57.214 ± 0.58	0.045^*^
Nasal	55.125 ± 0.87	56.225 ± 0.93	0.025^*^
Inferior	54.324 ± 1.33	55.575 ± 1.08	0.040^*^
Central foveal thickness	231.829 ± 4.2	244.829 ± 4.2	0.007^*^
Subfoveal choroidal thickness	319.821 ± 11.47	322.082 ±11.47	0.026^*^
Perimetry			
Mean defect (dB)	2.21 ± 1.21	0.93 ± 0.37	0.013^*^

Values are presented as the mean ± SD.

* = statistically significant.

CC= choriocapillaris; FAZ= foveal avascular zone; SD= standard deviation.

**Table 7:** Macular perfusion and perimetric parameters of the right eye and left eye in the HCQ group

	Right eye n=28	Left eye n = 28	P
Superficial retinal flow area (mm^2^)	13.468 ± 0.34	13.511 ± 0.81	0.964
Deep retinal flow area (mm^2^)	13.211 ± 0.38	13.607 ± 0.28	0.657
CC flow area (mm^2^)	17.573 ± 0.39	17.459 ± 0.37	0.843
FAZ area (superficial, mm^2^)	0.338 ± 0.011	0.330 ± 0.015	0.745
FAZ area (deep, mm^2^)	0.351 ± 0.010	0.362 ± 0.014	0.635
Superficial vessel density (%)			
Fovea	34.127 ± 1.44	34.531 ± 1.37	0.523
Parafoveal	53.613 ± 1.32	53.695 ± 1.39	0.631
Temporal	54.933 ± 0.88	54.954 ± 1.34	0.901
Superior	53.294 ± 1.25	53.137 ± 0.70	0.886
Nasal	53.524 ± 1.66	53.397 ± 1.22	0.832
Inferior	53.317 ± 0.99	53.328 ± 1.11	0.991
Deep vessel density (%)			
Fovea	36.112 ± 0.96	36.038 ± 0.87	0.874
Parafoveal	55.408 ± 1.22	55.507 ± 0.98	0.663
Temporal	54.119 ± 1.13	54.222 ± 1.24	0.658
Superior	54.953 ± 1.34	54.284 ± 1.18	0.503
Nasal	52.111 ± 0.98	54.862 ± 0.91	0.846
Inferior	52.779 ± 1.22	52.973 ± 1.35	0.542
Central foveal thickness	237.114 ± 5.5	237.004 ± 4.9	0.411
Subfoveal choroidal thickness	306.867 ± 9.8	308.222 ± 10.1	0.327
Perimetry			
Mean defect (dB)	2.88 ± 1.65	2.94 ± 1.35	0.203
Standard loss variance (dB)	1.87 ± 0.94	1.91 ± 0.97	0.855

Values are presented as the mean ± SD.

HCQ= hydroxychloroquine; CC= choriocapillaris; FAZ= foveal avascular zone; SD= standard deviation.

In patients treated with HCQ, OCTA imaging showed loss of the perifoveal photoreceptor inner segment/outer segment junction (three patients), perifoveal thinning of the outer nuclear layer (two patients), and an apparent posterior displacement of the inner retinal structures toward the RPE (one patient).

## DISCUSSION

HCQ-induced retinopathy is a clinical condition occurring in patients who receive >6.5 mg/kg daily dose, characterized by impairment in visual acuity and deterioration in visual field^(5)^. Risk factors for toxic retinopathy are >6.5 mg/kg daily dose; >1,000 g cumulative dose; >5 years of treatment; increased age (>60 years); concomitant liver or kidney dysfunction; and presence of any basal maculopathy^(6)^. The early detection of HCQ-induced retinal toxicity is important because of the risk of irreversible vision loss^(5)^.

The primary aim of this study is to evaluate the retinal vascular structure in patients who received HCQ. The secondary aim is to investigate whether OCTA is valuable in detecting HCQ-induced retinal toxicity. In this study, SCP (crucial for ganglion cell layer nutrition) and vascular density were evaluated as superficial retinal flow area and superficial vessel density, respectively. Both values were significantly decreased in the HCQ group. The outer retina and DCP, which consists of photoreceptors, were evaluated as deep retinal flow area and deep vessel density; these values were also significantly decreased in the HCQ group. Our analysis showed significantly decreased CC flow area and SFCT in the HCQ group compared with those in the control group. Both perimetric parameters (i.e., mean defect and standard loss variance values) were higher in the HCQ group. The FAZ area (superficial and deep) was significantly enlarged in the HCQ group compared with that in the control group. The results of this study revealed a significant deterioration in macular microvascular circulation in patients treated with HCQ.

Two previous reports evaluated the retinal microvascular structure by OCTA in patients treated with HCQ. Bulut et al.^(14)^ evaluated a total of 60 patients in two groups: a high-risk group (duration of treatment ≥5 years) and a low-risk group (duration of treatment <5 years). Both groups were evaluated for HCQ-induced retinal toxicity using the visual field test, OCTA, and spectral domain OCT (SD-OCT). The findings revealed that vascular density, retinal and choroidal flow rates, and choroidal thickness parameters were significantly decreased in the high-risk group compared with those in the low-risk group. However, the study conducted by Bulut et al.^(14)^ lacked a control group. In the present study, a control group was included, and the highand low-risk subgroups were further analyzed. Consistent with the findings reported by Bulut et al.^(14)^, the results obtained from the subgroup analyses in the present study revealed greater decrease in retinochoroidal flow and vascular density in the high-risk group. Our study evaluating the superficial and deep vascular plexus in the foveal, parafoveal, superior, inferior, temporal, and nasal regions revealed significant decrease in flow and vascular density in the HCQ group. In contrast, in the study conducted by Bulut et al.^(14)^, these parameters were evaluated as a whole (i.e., in the foveal and parafoveal areas, but not in the superior, inferior, nasal, or temporal regions). A study performed by Ozek et al.^(15)^ evaluated retinal toxicity in 40 patients who received HCQ for rheumatoid arthritis. The patients were assigned to high-and low-risk groups and compared with age-matched controls. Ozek et al.^(15)^ observed that the deep vascular density in the temporal and inferior regions was significantly lower in the high-risk group than that in the control group; nevertheless, these differences were not detected in the low-risk group. There was no significant difference observed in the density of the superficial vascular structure between the HCQ and control groups. Moreover, there was no significant difference between the high-and low-risk groups in terms of superficial and deep vascular density. However, we noted a significant decrease in both superficial and deep vascular densities in the HCQ group. These findings are in accordance with those of Ozek et al.^(15)^ for the deep vascular structure but not for the superficial vascular structure. Additionally, there is a disagreement between the two studies in terms of the findings in the highand low-risk groups. In the present study, we demonstrated significant impairment in macular microcirculation in the high-risk group. In contrast, Ozek et al.^(15)^ did not reveal a significant difference. This inconsistency may be attributed to the evaluation criteria. In the present study, we assessed macular perfusion using flow measurements and vascular density analysis. However, in the study conducted by Ozek et al., only vascular density was evaluated^(15)^.

We demonstrated a significant correlation between the retinochoroidal flow, vascular density, and the cumulative dose of HCQ; there was no significant correlation noted between the retinochoroidal flow, vascular density, and the duration of treatment with HCQ in accordance with the report by Bulut et al.^(14)^ Lyons et al.^(16)^ reported a significant correlation between the cumulative HCQ dose and multifocal ERG anomalies in their study comparing 67 patients treated with HCQ and 62 healthy controls. In a large group consisting of 3,995 HCQ-treated patients, Wolfe et al.^(3)^ reported that retinal toxicity induced by HCQ was significantly frequent in patients who received the treatment for >7 years with a cumulative dose of >1,000 g. Collectively, these results confirm the recommendation from the American Academy of Ophthalmology, indicating that the main determinants of retinal toxicity are the daily and cumulative doses^(3,17,18)^. In the present study, there was no significant correlation recorded between the duration of treatment and macular perfusion, which was compatible with the results of previous studies^(3,14,17,18)^.

Similar to the findings reported by Bulut et al.^(14)^, the FAZ area in our study was significantly enlarged in both the superficial and deep retinal layers and correlated with the daily and cumulative doses of HCQ. However, this finding was not observed by Ozek et al.^(15)^.

In a study evaluating choroidal vascular dysfunction through OCTA, Ahn et al.^(19)^ revealed a significant decrea se in choroidal thickness and CC equivalent thickness value that was correlated with the cumulative dose and body weight. The investigators observed that richly pigmented CC with thinner vessels is markedly more affected than large or medium calipered choroidal vessels. Bulut et al.^(14)^ reported a significant decrease in choroidal flow and thickness. Concordant with previous reports, our study revealed a significant decrease in both choroidal flow and thickness, suggesting choroidal vascular dysfunction that may be related to HCQ toxicity^(14,19)^.

The 10-2 visual field test requires the cooperation of the patients and is thus characterized by subjectivity. Therefore, it is more valuable to objectively evaluate the retinal toxicity of HCQ. We found a significant correlation between perimetric values (i.e., mean defect and standard loss variance), cumulative dose, duration of treatment, and retinochoroidal perfusion parameters (i.e., superficial retinal flow area, deep flow area, and CC). These findings support the positive correlation between the deterioration of the visual field and retinochoroidal flow and vascular density, similar to the study conducted by Bulut et al.^(14)^. Marmor et al.^(20)^ revealed a paracentral scotoma in 10% (11 patients) despite the lack of any pathological finding in SD-OCT in patients who received >6.5 mg/kg daily or cumulative >1,000 g dose for >9 years. Hence, they suggested to use the visual field test in conjunction with SD-OCT. Chen et al.^(21)^ reported that nine of 25 patients had fundus pathologies. However, four of those patients had normal SD-OCT and visual field findings, and one patient had normal SD-OCT findings despite visual field defects. Although they reported visual field defects in eight patients, only one of those had pathological SD-OCT findings. The study conducted by Chen et al.^(21)^ indicated that neither the visual field test nor SD-OCT individually is capable of detecting retinal toxicity induced by HCQ. In our study, evaluation using an objective measurement technique showed a correlation between the visual field parameters and retinochoroidal flow and a decrease in vascular density. This approach offers valuable data regarding the usage of OCTA as an objective complementary test in patients treated with HCQ. Considering that the probability of experiencing an adverse effect related to HCQ is 6.5% and the discontinuation rate of HCQ due to retinal toxicity is 1.8%, the determination of HCQ-induced retinal toxicity becomes increasingly important^(3)^. Accumulation of HCQ in the RPE has been well documented in previous studies^(1)^. OCTA could not demonstrate these deposits in the RPE layer; therefore, our OCTA findings concerning vascular damage attributed to HCQ toxicity in this study may serve as an adjunctive indirect marker rather than a direct indicator of HCQ toxicity. Furthermore, OCTA may be an alternative approach to the rapid and objective measurement of the macular flow in uncooperative patients who are incapable of confidently answering the visual field test.

Central retinal thickness was significantly reduced and negatively correlated with the HCQ dose in our study. Bulut et al.^(14)^ did not observe any significant difference between the lowand high-risk groups with regard to central macular thickness in OCTA measurements. This discrepancy may be due to the inclusion of healthy controls in the present study. Ozek et al.^(15)^ determined that retinal thickness was significantly reduced in the temporal and inferior parafoveal areas in both the lowand high-risk groups versus those in the control group. Yulek et al.^(22)^ reported that parafoveal retinal thickness was significantly decreased at 6 months post treatment compared with the pretreatment measurements by SD-OCT in 46 newly diagnosed and HCQ-treated patients. Notably, the perifoveal retinal thickness, ganglion cell complex, and retinal nerve fiber layer did not change. Yulek et al.^(22)^ revealed that HCQ toxicity occurred mostly in the central parafoveal retina. It was especially significant in the superior, nasal, and temporal areas but not significant in the inferior parafoveal area. Using an adaptive optics camera that enables the evaluation of the photoreceptor layer, Babeau et al.^(23)^ showed that HCQ-induced retinal toxicity in 38 HCQ-treated patients was significantly correlated with the daily dose and cumulative dose, especially in the inferior parafoveal area. Marmor et al.^(17)^ reported that initial signs of HCQ-induced retinal toxicity were first detected in the inferior parafoveal area. In our study, the daily and cumulative doses were correlated with the superficial and deep vascular densities in all areas. Furthermore, daily dose and cumulative dose were highly correlated (r>-0.7) with the inferior area and poorly correlated (r<-0.2) with the whole other areas vessel density, in accordance with the previous studies indicating the localization of the retinal toxicity^(15,17,23)^. The correlation between the initial inferior parafoveal area of retinal damage and vessel density may lead to further studies for the early detection of retinal toxicity.

Fluorescein angiography (FA) is an established invasive imaging method. This technique requires the use of a dye, which is associated with the occurrence of adverse effects^(24)^. Although FA is useful for visualizing the retinal vasculature, its inability to show the distinct vascular structures of the different retinal layers may be a shortcoming in comparison with OCTA. OCTA allows the independent examination of the superficial and deep vascular plexi. Therefore, it may reveal early changes in the vascular tissue that arise in the nascent stages of certain diseases of vascular origin (e.g., diabetes) earlier than FA^(25)^. In addition, visualization of the deep retinal vascular plexus is not possible with FA. Ozek et al.^(15)^ demonstrated vascular signs of HCQ toxicity only in the deep vascular plexus, which cannot be visualized using FA. We propose that HCQ-related vascular damage can be detected earlier and localized more effectively with OCTA versus FA.

The limitations of this study were the relatively small sample size and cross-sectional design. We are currently examining a larger sample and planning to present our data regarding long-term outcomes in the future. Longitudinal studies are warranted to determine the predictive value and clinical importance of such findings (especially the inferior area vessel density) in the screening of HCQ-induced maculopathy. Data of five patients who had deterioration of inner retinal layers were compared with those of other patients. Additionally, there are only two other studies in the literature that evaluated HCQ-induced retinal toxicity by measuring the macular microcirculation via OCTA. Thus, our findings need to be confirmed by other studies. Although rheumatic diseases are associated with vascular pathologies, our study was not homogeneous in terms of the presence of systemic diseases. This heterogeneity may also be a limitation of our study. Patients in the HCQ group had a significantly lower retinal thickness than healthy controls. Some researchers have reported that retinal thinning may significantly alter the retinal segmentation in SCP and DCP and cause errors in automatic calculations^(26)^. The choroid was measured using OCTA, which functions very poorly in visualizing the retinochoroidal interface and may significantly affect the reliability of the results. This should be taken into account in our statements regarding retinal and choroidal thickness or vascular density.

Early detection of HCQ-induced toxicity is crucial to avoid permanent retinal damage. However, achieving this aim through the use of only one monitoring modality may be difficult. We suggest that a combination of OCT, OCTA, visual field, and multifocal ERG tests within the capability of the clinic may offer earlier detection of HCQ-induced retinal toxicity. The OCTA approach provided objective macular perfusion measurements and revealed correlations between the cumulative and daily doses and between the inferior parafoveal deterioration and HCQ-induced retinal toxicity. Hence, this method may be useful as a complementary technique to visual field analysis and other monitoring techniques for HCQ-induced retinal toxicity.
